# Paternal weight prior to conception and infant birthweight: a prospective cohort study

**DOI:** 10.1038/s41387-021-00172-1

**Published:** 2021-09-14

**Authors:** Ravi Retnakaran, Shi Wu Wen, Hongzhuan Tan, Shujin Zhou, Chang Ye, Minxue Shen, Graeme N. Smith, Mark C. Walker

**Affiliations:** 1 Leadership Sinai Centre for Diabetes, Mount Sinai Hospital, Toronto, ON Canada; 2 Division of Endocrinology, University of Toronto, Toronto, ON Canada; 3 Lunenfeld-Tanenbaum Research Institute, Mount Sinai Hospital, Toronto, ON Canada; 4 OMNI Research Group, Department of Obstetrics and Gynecology, University of Ottawa, Ottawa, ON Canada; 5 Ottawa Hospital Research Institute, Clinical Epidemiology Program, ON Ottawa, Canada; 6 Department of Epidemiology and Community Medicine, University of Ottawa, Ottawa, ON Canada; 7 School of Public Health, Central South University, Changsha, China; 8 Liuyang Municipal Hospital of Maternal and Child Health, Beizheng, Liuyang, China; 9 Queen’s Perinatal Research Unit, Department of Obstetrics and Gynecology, Queen’s University, Kingston, ON Canada

**Keywords:** Risk factors, Epidemiology

## Abstract

**Background/Objective:**

Previous studies have consistently demonstrated that maternal weight status both before and during pregnancy is associated with infant birthweight. However, a fundamental limitation across this literature remains that previous studies have not evaluated the concomitant impact of paternal weight at conception, owing to the paucity of studies in which fathers were assessed prior to pregnancy. Thus, we established a cohort of preconception couples to prospectively evaluate the associations of maternal and paternal weight prior to pregnancy with infant birthweight at delivery.

**Methods:**

In this prospective observational cohort study, 1292 newly-married women and their partners in Liuyang, China, were assessed at median of 23.3 weeks before a singleton pregnancy, thereby enabling concomitant assessment of preconception maternal and paternal body mass index (BMI) in relation to infant birthweight.

**Results:**

Mean birthweight was 3294 ± 450 g with 147 neonates (11.4%) born large-for-gestational-age (LGA) and 94 (7.3%) small-for-gestational-age (SGA). After adjustment for maternal and paternal factors prior to conception (age, education, smoking, BMI, household income), length of gestation, total gestational weight gain, gestational diabetes, preeclampsia, and infant sex, it was noted that infant birthweight increased by 42.2 g (95% CI 29.5–54.8; *p* < 0.0001) per unit increase in maternal pregravid BMI and 10.7 g (95% CI 0.5–20.9; *p* = 0.04) per unit increase in paternal pregravid BMI. Maternal pregravid BMI explained 6.2% of the variance in birthweight whereas paternal BMI explained only 0.7%. Independent predictors of LGA delivery were maternal pregravid BMI (aOR = 1.91, 95% CI 1.50–2.44), maternal age (aOR = 1.48, 95% CI 1.09–2.00), and gestational weight gain (aOR = 1.80, 95% CI 1.40–2.30). Paternal pregravid BMI was not independently associated with LGA or SGA.

**Conclusion:**

Paternal BMI prior to conception is associated with infant birthweight but only modestly so, in contrast to the dominant impact of maternal weight.

## Introduction

Infant birthweight is a neonatal outcome that holds both short- and long-term implications. In the short-term, at delivery, macrosomia is associated with increased risks of shoulder dystocia, birth injury, and the need for Cesarean section [1]. Over the long-term, individuals born with low birthweight (and to a lesser degree those with high birthweight) have elevated risks of developing type 2 diabetes and coronary artery disease several decades later in adulthood [2, 3]. The presumed mechanistic basis underlying these long-term associations is the Developmental Origins of Health and Disease (DOHaD) paradigm, which posits that intrauterine exposures program developmental pathways in the fetus that affect both birthweight and later-life trajectories of cardiometabolic risk [2]. Accordingly, given these implications, investigation of the determinants of birthweight is a well-established focus of research interest.

In this context, previous studies have consistently identified maternal weight status both at conception and during pregnancy as a key determinant of infant birthweight [4–6]. However, a fundamental limitation across this literature remains that previous studies have not evaluated the concomitant impact of paternal weight at conception [7–10]. Though paternal weight at conception is recognized as a factor of great interest, its evaluation in previous studies has been precluded by the logistical challenge of identifying, recruiting, and assessing couples just prior to pregnancy. Thus, to address this critical knowledge gap, we have established a cohort of preconception couples to prospectively evaluate the associations of pregravid maternal and paternal weight with infant birthweight.

## Methods

For this study, we constructed a prospective preconception observational cohort, in which couples in the Liuyang region of Hunan province in China were recruited at the time of marriage. In this cohort, participating women and their partners underwent baseline (pregravid) evaluation at recruitment and then, whenever they subsequently became pregnant, were followed across gestation to delivery [11, 12]. The study was approved by the institutional research ethics boards of Central South University (Changsha, Hunan, China), Ottawa Hospital Research Institute (Ottawa, Canada), and Mount Sinai Hospital (Toronto, Canada), and all participants provided written informed consent.

### Construction of preconception cohort

To establish a preconception cohort in a cost-efficient manner, it is necessary to have a way of identifying women who are likely to become pregnant in the near future. This opportunity exists in Liuyang because women in this region attend a premarriage health clinic for assessment at the time of marriage registration. Thus, to establish this cohort, we recruited women from these clinics at Liuyang Maternal and Infant Hospital, if they indicated an intention to conceive in the next 6 months. The partners of participating women were also approached for participation. Beginning in February 2009, 3375 women were recruited into a maternal cohort, of whom 2382 have completed a singleton pregnancy. Amongst these women, 1519 male partners underwent baseline pregravid evaluation. After the exclusion of 40 women who were >5 weeks pregnant at their baseline assessment based on back-dating of gestational age at delivery and 187 women with incomplete infant delivery data, the study population for the current analysis consisted of 1292 couples who completed their baseline visit prior to a singleton pregnancy (Online Fig. 1). At the baseline visit, both parents completed interviewer-administered questionnaires and underwent physical examination included assessment of blood pressure and anthropometry (weight, height, and waist circumference) by trained study staff. Blood pressure was measured in a seated position, after 10 min of rest, using an automated noninvasive blood pressure monitor, with two measurements performed 10 min apart and the average recorded. Height and weight were measured with a wall-mounted stadiometer and scale, with both measurements performed twice and the average recorded for each. Waist circumference was measured at the midpoint between the lower edge of the ribs and the iliac crest, with two measurements performed and the average recorded.

### Assessment at delivery

Data collected at delivery included gestational age (based on last menstrual period), total weight gain in pregnancy (weight was measured at each clinical visit in pregnancy and prior to delivery), major maternal medical complications in pregnancy (including gestational diabetes mellitus and preeclampsia), infant sex, birthweight, and mode of delivery. At the participating hospital, all pregnant women undergo a 2-h 75 g oral glucose tolerance test as screening for gestational diabetes. Gestational diabetes was diagnosed on the oral glucose tolerance test if one of the following thresholds were met: fasting glucose ≥5.1 mmol/l; 1-h glucose ≥10.0 mmol/l; or 2-h glucose ≥8.5 mmol/l. Preeclampsia was diagnosed based on blood pressure ≥140/90 mmHg and 24-h urine protein >0.3 g or positive random urine protein, at ≥20 weeks gestation. Large-for-gestational-age (LGA) was defined as birthweight for gestational age above the 90th percentile according to birthweight centiles for a Chinese population [13]. Small-for-gestational-age (SGA) was defined as birthweight for gestational age below the tenth percentile on birthweight centiles for a Chinese population [13].

### Statistical analyses

All analyses were performed using SAS 9.4 (SAS Institute, Cary, NC). Continuous variables were tested for normality of distribution, with natural log-transformation applied where necessary for skewed variables. The study population was first stratified into tertiles based on infant birthweight, enabling the comparison of maternal and paternal characteristics between these groups (Table 1). Continuous variables were compared using analysis of variance for those that were normally distributed and Wilcoxon rank-sum nonparametric test for those that were skewed. Categorical variables were compared by chi-square or Fisher’s exact test.

**Table 1:** Characteristics of study population before pregnancy and at delivery, stratified by tertile of infant birthweight. Continuous data are presented as mean ± SD (if normally distributed) or median followed by interquartile range in parentheses (if skewed).

	Lowest tertile	Middle tertile	Highest tertile	
	[1250–3060 g]	[3100–3450 g]	[3500–6000 g]	
Pregravid characteristics	*n* = 372	*n* = 478	*n* = 442	*P*
Age:				
Maternal (yrs)	24 ± 3	25 ± 3	25 ± 3	0.01
Paternal (yrs)	26 ± 3	26 ± 3	26 ± 4	0.06
Years of education:				
Maternal (yrs)	9 (9–12)	11 (9–12)	12 (9–12)	0.08
Paternal (yrs)	9 (9–12)	9 (9–12)	9 (9–12)	0.17
Household income (1000 yuan)	20 (10–30)	20 (19–30)	20 (12–30)	0.004
Smoking:				
Maternal (*n* (%))	0 (0)	1 (0.2)	5 (1.2)	0.03
Paternal (*n* (%))	180 (51.3)	195 (45.1)	178 (43.6)	0.09
BMI:				
Maternal (kg/m^2^)	19.7 ± 2.3	20.1 ± 2.4	20.6 ± 2.6	<0.0001
Paternal (kg/m^2^)	21.6 ± 2.6	22.3 ± 2.7	22.0 ± 2.6	0.0008
Waist:				
Maternal (cm)	68.8 ± 7.7	70.7 ± 7.6	72.0 ± 7.3	<0.0001
Paternal (cm)	74.5 ± 8.1	75.7 ± 8.0	75.9 ± 8.1	0.02
**At delivery**				
Length of gestation (weeks)	38 ± 2	39 ± 1	39 ± 1	<0.0001
Total gestational weight gain (kg)	15.0 ± 6.1	16.7 ± 6.5	18.8 ± 7.0	<0.0001
Gestational diabetes (*n* (%))	5 (1.3)	7 (1.5)	14 (3.2)	0.10
Preeclampsia (*n* (%))	11 (3.0)	4 (0.8)	4 (0.9)	0.03
Cesarean delivery (*n* (%)	124 (33.7)	148 (31.2)	201 (45.9)	<0.0001
Male infant (*n* (%))	167 (44.9)	240 (50.2)	266 (60.2)	<0.0001
Birthweight (g)	2782 ± 267	3262 ± 112	3759 ± 286	<0.0001
LGA (*n* (%))	1 (0.3)	0 (0)	146 (33.0)	<0.0001
SGA (*n* (%))	94 (25.3)	0 (0)	0 (0)	<0.0001

Multiple linear regression analysis was conducted to identify independent determinants of infant birthweight (Table 2). Covariates in this model included maternal and paternal factors prior to conception (age, years of education, smoking status, BMI, and household income), and antepartum factors that are known or expected to influence birthweight (length of gestation, total gestational weight gain, gestational diabetes, preeclampsia, and infant sex). Since the study population consisted of newly-married couples wherein paternal BMI and maternal BMI were not strongly associated (Spearman correlation coefficient *r* = 0.05, *p* = 0.087), we included both paternal and maternal BMI in the model. A simple linear regression of each covariate was also conducted in parallel to determine the unadjusted association with infant birthweight. Furthermore, to explain the comparative associations of maternal and paternal BMI before conception on subsequent infant birthweight, we used partial *R*^2^ to measure the contribution of maternal and paternal BMI to the variance of infant birthweight, when controlling other covariates.

**Table 2:** Change in infant birthweight in relation to pregravid and pregnancy factors. Data were presented as the change in birthweight per unit change in the indicated variable, either unadjusted (crude) or after adjustment for all of the other listed variables.

	Change in infant birthweight (g)		
Variables	Crude (95% CI)	Adjusted (95% CI)*	P**
Maternal age (yrs)	14.8 (6.8 to 22.8)	20.3 (8.2 to 32.5)	0.001
Paternal age (yrs)	7.0 (−0.2 to 14.3)	−2.2 (−13.6 to 9.3)	0.71
Maternal education (yrs)	11.2 (1.0 to 21.5)	5.6 (−9.8 to 20.9)	0.47
Paternal education (yrs)	6.8 (−3.2 to 16.8)	6.4 (−7.8 to 20.6)	0.38
Household income (1000 yuan)	2.5 (1.0 to 4.0)	0.7 (−1.4 to 2.8)	0.52
Maternal smoking	516.5 (157.2 to 875.8)	138.3 (−413.0 to 689.6)	0.62
Paternal smoking	−43.6 (−95.6 to 8.4)	−20.4 (−80.1 to 39.4)	0.50
Maternal pregravid BMI (kg/m^2^)	28.9 (18.0 to 39.8)	42.2 (29.5 to 54.8)	<0.0001
Paternal pregravid BMI (kg/m^2^)	11.3 (2.1 to 20.5)	10.7 (0.5 to 20.9)	0.04
Length of gestation (weeks)	142.3 (125.7 to 158.8)	137.2 (115.0 to 159.3)	<0.0001
Total gestational weight gain (kg)	16.1 (12.1 to 20.2)	18.0 (13.5 to 22.4)	<0.0001
Gestational diabetes	251.9 (77.2 to 426.5)	92.1 (−127.2 to 311.4)	0.40
Preeclampsia	−338.4 (−541.9 to −135.0)	−236.0 (−479.0 to 7.0)	0.06
Male infant	133.6 (84.9 to 1852.3)	174.0 (114.7 to 233.3)	<0.0001

*Adjusted for all other variables listed. The adjusted estimates can be interpreted in the following way: Infant birthweight increased by 137.2 g per additional week of gestation, after adjustment all of the other variables.

**P value refers to estimate for indicated variable in the adjusted model.

**Fig. 1:**
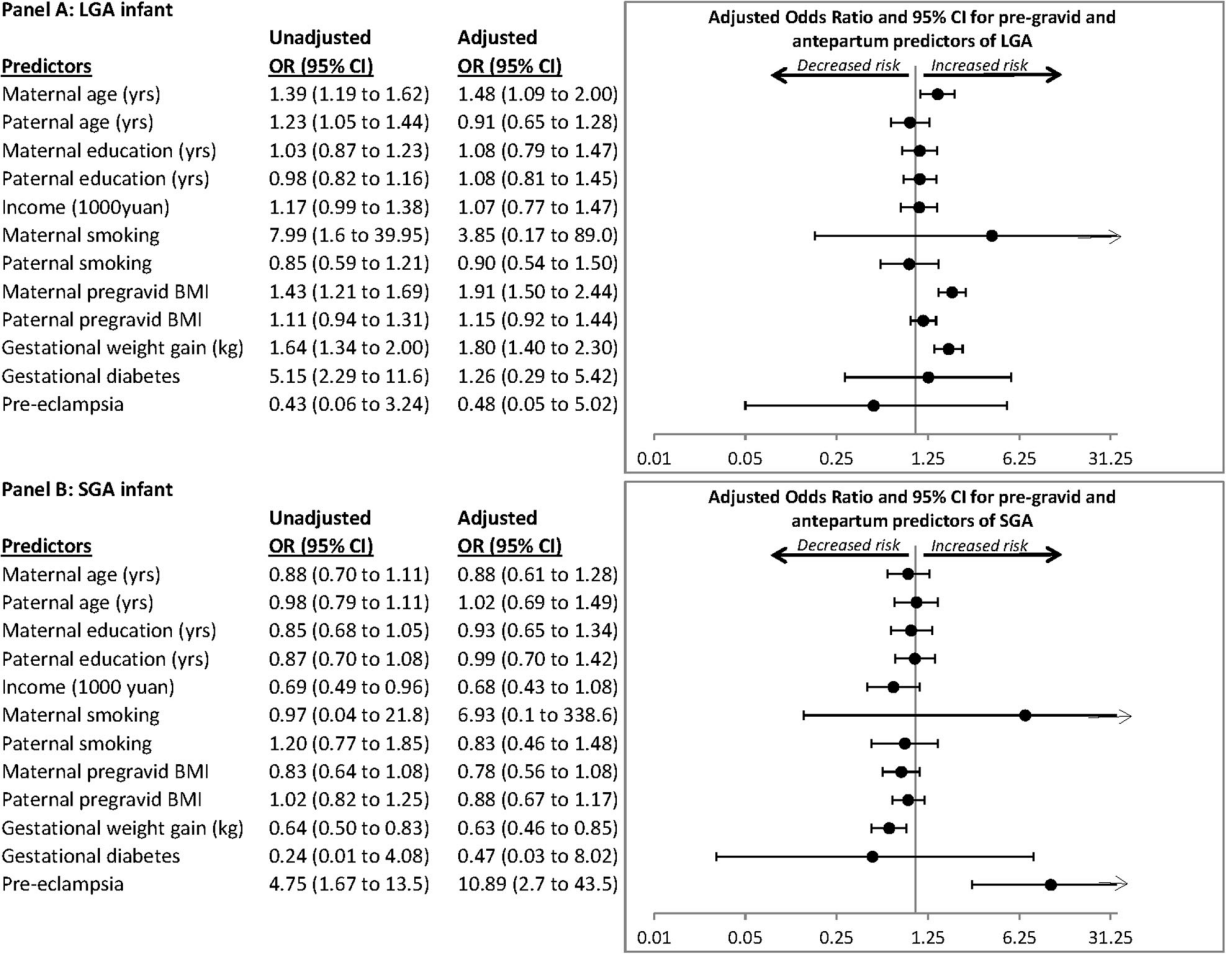
Predictors of large-for-gestational age and small-for-gestational age. Pregravid and antepartum predictors of (**A**) delivering a large-for-gestational-age (LGA) infant and (**B**) delivering a small-for-gestational-age (SGA) infant. Data were presented as odds ratio per standard deviation change in the indicated variable, either unadjusted or after adjustment for all of the other listed variables.

Multiple logistic regression analysis was conducted to identify independent determinants of LGA (Fig. 1A). This model included the same covariates as the multiple linear regression of birthweight, with the exception of the length of gestation and infant sex (both of which are incorporated into the definition of LGA). A simple logistic regression was also applied to each covariate to examine its unadjusted association with LGA. The same approach was applied to logistic regression analysis of SGA, with the same covariates as those for the LGA model (Fig. 1B).

## Results

The study population consisted of 1292 newly-married women and their partners, who were assessed at median of 23.3 weeks before a singleton pregnancy. At delivery, the mean infant birthweight was 3294 ± 450 g, with 147 neonates (11.4%) born LGA and 94 (7.3%) SGA. Table 1 shows the characteristics of the study population, stratified into three groups based on tertiles of infant birthweight. At the pregravid assessment, maternal BMI and paternal BMI both increased progressively across the groups from the lowest to the middle to highest tertile of infant birthweight (*p* < 0.0001 and *p* = 0.0008, respectively). The same pattern was observed for maternal and paternal waist circumference (*p* < 0.0001 and *p* = 0.02, respectively), maternal age (*p* = 0.01), and household income (*p* = 0.004). At delivery, the prevalence of male infant and Cesarean delivery increased from the lowest to the highest tertile of birthweight (both *p* < 0.0001), coupled with longer length of gestation and greater gestational weight gain (both *p* < 0.0001). In addition, the prevalence of preeclampsia was highest in the lowest tertile of birthweight (*p* = 0.03).

We next performed multiple linear regression analysis to identify independent determinants of infant birthweight (Table 2). In a model that explained 33.2% of the variance in birthweight, both maternal pregravid BMI and paternal pregravid BMI emerged as significant predictors of this outcome (*p* < 0.0001 and *p* = 0.04, respectively). Other significant predictors of birthweight were total gestational weight gain (*p* < 0.0001), length of gestation (*p* < 0.0001), male infant (*p* < 0.0001), and maternal age (*p* = 0.001). Paternal age was not significantly associated with birthweight.

We next performed a series of sensitivity analyses. First, when we excluded the 52 infants with a length of gestation <37 weeks, the significant predictors of birthweight were unchanged, including maternal pregravid BMI (*p* < 0.0001), paternal pregravid BMI (*p* = 0.03), total gestational weight gain (*p* < 0.0001), length of gestation (*p* < 0.0001), male infant (*p* < 0.0001), and maternal age (*p* = 0.0006) (data not shown). Second, when the model included paternal pregravid weight and height in place of BMI, paternal weight (*p* = 0.04), but not height (*p* = 0.43), was associated with infant birthweight, while the other significant predictors were unchanged (data not shown). Third, a sensitivity analysis in which pregravid maternal and paternal BMI were replaced with the analogous pregravid parental waist circumference measurements yielded similar findings to those in Table 2 except that only maternal but not paternal waist was associated with birthweight (*p* < 0.0001 and *p* = 0.15, respectively) (data not shown). Finally, when the multiple linear regression analysis was limited to the 506 pregnancies in which either the father or the mother had pregravid overweight (defined by BMI ≥23 kg/m^2^), the findings were again similar, with only maternal BMI (*p* < 0.0001) but not paternal BMI (*p* = 0.13) associated with birthweight (data not shown).

To further elucidate the comparative associations of maternal and paternal BMI before conception on subsequent infant birthweight, we returned to the multiple linear regression model in Table 2. When this model did not include paternal BMI, maternal pregravid BMI explained 6.2% of the variance in birthweight. Upon the addition of paternal BMI to the model, the proportion of the variance explained by maternal pregravid BMI was unchanged (6.2%), while paternal BMI reconciled only 0.7%. Thus, while paternal pregravid BMI is significantly associated with infant birthweight, its impact is modest, as compared to that of maternal prepregnancy weight.

On logistic regression analysis, the significant predictors of having an LGA infant were maternal pregravid BMI (aOR = 1.91, 95% CI 1.50–2.40), maternal age (aOR = 1.48, 95% CI 1.09–2.00), and total gestational weight gain (aOR = 1.80, 95% CI 1.40–2.30) (Fig. 1A). Neither paternal BMI nor paternal age prior to conception was significantly associated with LGA. These findings were unchanged when pregravid maternal and paternal BMI were replaced with the analogous pregravid parental waist circumference measurements (data not shown). The only significant predictors of having an SGA infant were total gestational weight gain (aOR = 0.63, 95% CI 0.46–0.85) and preeclampsia (aOR = 10.89, 95% CI 2.7–43.5) (Fig. 1B). Neither maternal nor paternal BMI prior to conception was associated with SGA.

## Discussion

In this study, infant birthweight increased by 42.2 g per unit increase in maternal pregravid BMI but by only a 10.7 g per unit increase in paternal pregravid BMI. Indeed, maternal pregravid BMI explained 6.2% of the variance in birthweight whereas paternal BMI explained only 0.7%. Moreover, maternal BMI before pregnancy (but not paternal) was a significant independent predictor of LGA. It thus emerges that paternal BMI prior to conception is modestly associated with infant birthweight, in contrast to the dominant impact of maternal pregravid weight.

While previous studies have consistently demonstrated that maternal weight before pregnancy is associated with infant birthweight [4, 5], it is important to recognize that the estimated association is typically biased by two factors. First, most studies do not have a direct measurement of maternal pregravid weight. Instead, because these studies typically recruit women during pregnancy, they either treat a first trimester weight measurement as a surrogate for pregravid weight or rely upon maternal self-report [14]. Second, previous reports have not accounted for the potential impact of paternal weight prior to conception [7–10]. Though some studies have measured paternal weight during pregnancy or at delivery [15, 16], the optimal design for addressing both of the limitations is a direct measurement of maternal and paternal anthropometry prior to conception, as in the current study.

The potential importance of considering maternal and paternal weight at the time of conception has been underscored by recognition of the DOHaD paradigm and, most recently, the emergence of the related concept of the Paternal Origins of Health and Disease (POHaD) [10, 17]. The POHaD concept holds that paternal factors (such as adiposity) can affect male germ cells, leading to their epigenetic modification, which has emerged as a potential mechanism by which the father’s weight at conception may influence the future health of his offspring [9, 10, 17–19]. Indeed, a growing body of preclinical animal data has suggested that paternal obesity alters the sperm epigenome, which transmits these epigenetic changes to the progeny, in whom metabolism is then altered [20–23]. Similarly, in humans, the sperm epigenome of obese men shows an altered DNA methylation profile (as compared to that of lean men) that has also been documented in the offspring of these men [24–27]. Thus, the POHaD concept highlights the potential importance of considering paternal BMI around the time of conception in relation to offspring outcomes such as birthweight.

Against this background, the current preconception cohort has provided the unique opportunity to prospectively evaluate the associations of maternal and paternal weight measured at a median of 23.3 weeks before pregnancy with subsequent infant birthweight. With this design, we demonstrate that pregravid maternal BMI and paternal BMI are both independently associated with birthweight, but with very different magnitudes of apparent effect (a concept that has been long recognized in the animal husbandry literature, as described in 1938 by Walton and Hammond in showing the dominant maternal effect in the crossbreeding of Shire horses and Shetland ponies [28]). Theoretically, both pregravid maternal weight and pregravid paternal weight could influence infant birthweight through inherited genetic factors, epigenetic effects, or shared environmental factors such as lifestyle practices, diet, and behavior. Notably, these effects could be similarly attributed to either parent. Conversely, the observed dominant effect of pregravid maternal BMI (as compared to paternal) potentially may point to a maternal-specific effect such as the impact of maternal adiposity on the intrauterine environment, though this remains conjecture at this time. It should also be recognized that the comparatively modest association between pregravid paternal BMI and birthweight does not necessarily rule out the possibility of a POHaD effect on offspring weight that may emerge later in childhood.

A key strength of this study is the prospective assessment of a large population of women and men just prior to pregnancy. Accordingly, this study design can provide an unbiased estimate of the independent associations of pregravid maternal and paternal BMI with infant birthweight. Conversely, given its observational nature, this design cannot provide evidence that modification of parental pregravid weight can affect birthweight. Another limitation is that the study population was comprised of a single ethnicity (Chinese) and was generally lean, such that these findings warrant replication in other populations of different ethnicities and body habitus types. However, as described earlier, Liuyang was specifically selected as the setting for this study because features of the local environment (single maternity hospital and premarriage health assessment) made it possible to establish the large cohort of preconception couples that is required for this analysis.

In quantifying the respective effects of maternal and paternal BMI before pregnancy on infant birthweight, the current data highlight the potential importance of maternal pregravid weight status for reducing the risk of fetal overgrowth and LGA delivery. These findings thus support recent calls for a clinical emphasis on optimizing maternal weight before conception [29, 30]. It should also be recognized that, although these data indicate that pregravid paternal BMI has only a modest impact on birthweight, further studies are needed to determine if effects on offspring growth and development may emerge later in childhood, as per the POHaD concept. For example, in the EDEN (Etude des Déterminants pré et postnatals précoces du développement et de la santé de l’ENfant) mother–child cohort, paternal BMI measured in the late second trimester of pregnancy was not associated with neonatal anthropometry at birth but showed subsequent association with infant weight at 3 months of age [31]. Similarly, in a study of 547 pregnancies, paternal BMI at 28 weeks of gestation was not associated with infant birthweight but correlated with offspring BMI at 1 year [32]. Thus, in light of the association between paternal pregravid BMI and infant birthweight observed in the current study, the evolution of this relationship over time with changes in offspring anthropometry during childhood warrants evaluation in future studies.

In conclusion, this preconception cohort study shows that infant birthweight increased by 42.2 g per unit increase in maternal pregravid BMI but by only a 10.7 g per unit increase in paternal pregravid BMI. While maternal pregravid BMI explained 6.2% of the variance in birthweight, paternal pregravid BMI explained only 0.7%. Furthermore, only maternal BMI before pregnancy was a significant independent predictor of LGA. It thus emerges that, while the prepregnancy BMI of each parent is independently associated with infant birthweight, the dominant influence is maternal weight, thereby supporting current calls for clinical focus on optimizing maternal weight status prior to pregnancy.

## Supplementary information

Online Figure
