# Supplementary figures and images for: Paternal weight prior to conception and infant birthweight: a prospective cohort study
Source: Nutr Diabetes. 2021 Sep 14;11:28. doi: 10.1038/s41387-021-00172-1 (PMC8440552; doi:10.1038/s41387-021-00172-1)

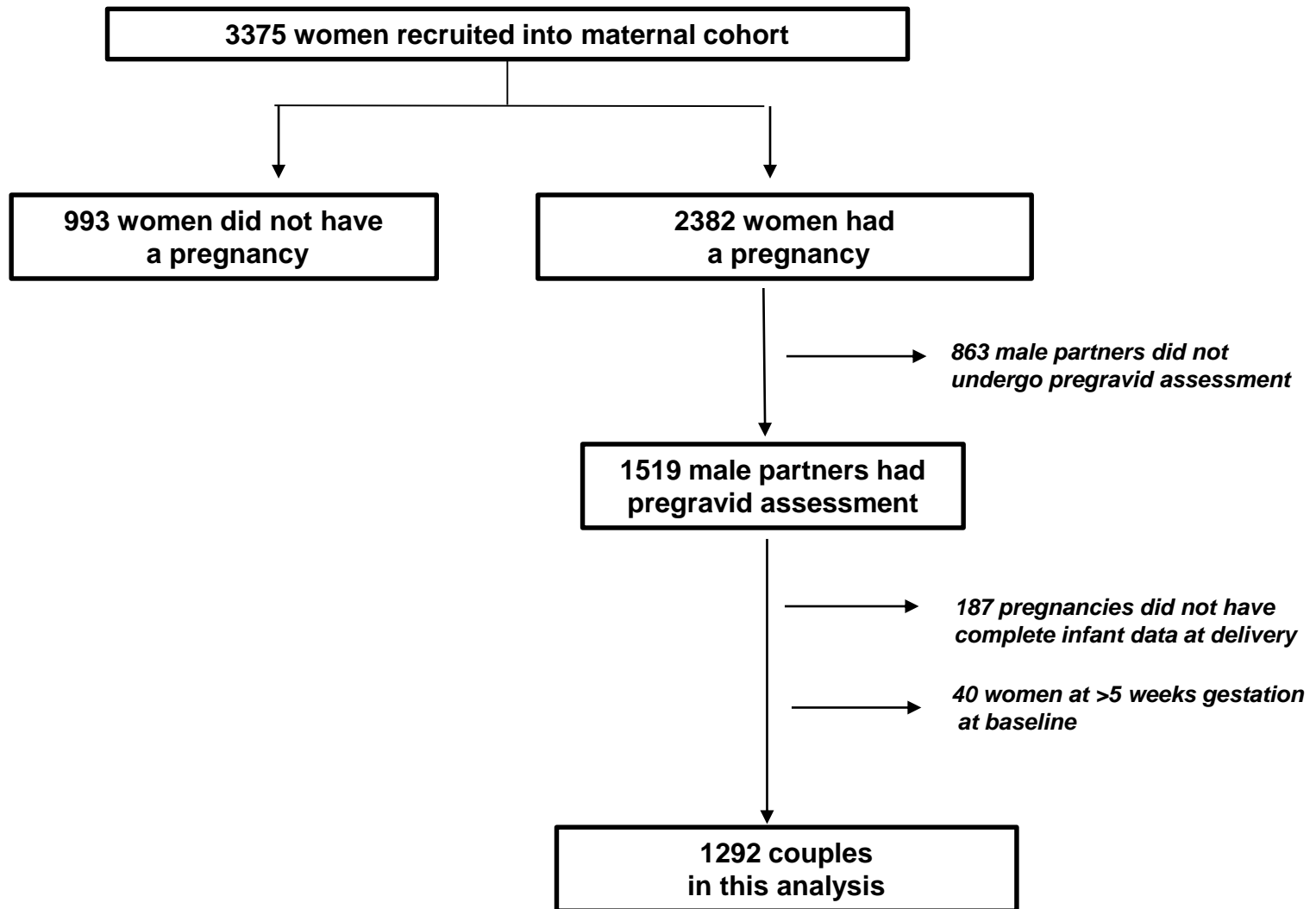

Online Figure 1: Derivation of study population

Supplement: Supplementary file 1 — Online Figure [file 41387_2021_172_MOESM1_ESM.pdf]
